# Role of Topotecan in Non-Small Cell Lung Cancer: A Review of Literature

**DOI:** 10.14740/wjon950e

**Published:** 2015-10-26

**Authors:** Adarsh Vennepureddy, Jean-Paul Atallah, Terenig Terjanian

**Affiliations:** a Department of Medicine, Staten Island University Hospital, Staten Island, NY 10305, USA; b Division of Hematology and Oncology, Staten Island University Hospital, Staten Island, NY 10305, USA

**Keywords:** Non-small cell lung cancer, Topotecan, Clinical trials, Tumor response

## Abstract

Topotecan (TPT), a chemotherapeutic agent, is a topoisomerase-I inhibitor. Topoisomerase-I is a nuclear enzyme that relieves torsion strain in DNA by opening single strand breaks which helps in DNA replication. TPT inhibits this enzyme, thus preventing DNA replication and causes cell death. TPT has demonstrated to have broad spectrum of antitumor activity in tumors like cervical, ovarian, endometrial and small cell lung cancers (SCLCs). The intravenous (IV) formulation of the drug is currently approved by the US Food and Drug Administration for the treatment of patients with SCLC and ovarian cancer at a dose of 1.5 mg/m^2^ administered daily for five consecutive days, with treatment cycles repeated every 3 weeks. TPT has shown some promising activity in the treatment of non-small cell lung cancer (NSCLC) with favorable side effect profile. Several clinical trials have been conducted with TPT in either IV or oral formulation for the treatment of NSCLC as a first or second-line treatment. Here we reviewed all the clinical trials done with TPT to date in the treatment of NSCLC both as a single-agent and combination therapy.

## Introduction

Topotecan (TPT), a chemotherapeutic agent, is a topoisomerase-I inhibitor. It is a water soluble derivative of camptothecin. Topoisomerase-I is a nuclear enzyme that relieves torsion strain in DNA by opening single strand breaks which helps in DNA replication. TPT inhibits this enzyme, thus preventing DNA replication and causes cell death [1-3].

TPT has demonstrated to have broad spectrum of antitumor activity in tumors like cervical, ovarian, endometrial and small cell lung cancers (SCLCs). The intravenous (IV) formulation of the drug is currently approved by the US Food and Drug Administration (FDA) for the treatment of patients with SCLC and ovarian cancer at a dose of 1.5 mg/m^2^ administered daily for five consecutive days, with treatment cycles repeated every 3 weeks. An oral formulation of TPT was also approved by the FDA in October 2007 for the treatment of relapsed SCLC [4]. TPT has shown some promising activity in the treatment of non-small cell lung cancer (NSCLC) with favorable side effect profile. However, TPT is not used as a first line of choice in the treatment of advanced NSCLC.

Lung cancer remains one of the leading causes of cancer-related death in men and women worldwide and attributes approximately 1.37 million deaths per year worldwide. NSCLC is the most common form of lung cancer and approximately 2/3 of patients with NSCLC present with advanced disease. This advanced disease state leads to limited treatment options, primarily systemic therapy [5]. Surgical resection offers the best opportunity for long-term survival and cure in patients with resectable NSCLC. The molecular characterization of tumor tissue (mutations in the EGFR, ALK, K-RAS genes) in patients with NSCLC serves as a guide to treatment both in those who present with metastatic disease and in those who relapse after primary therapy.

Combination cytotoxic chemotherapy with a platinum-based doublet is the backbone of the initial systemic treatment for patients with advanced NSCLC whose tumor does not have a driver mutation [5]. Combination chemotherapy regimens using a platinum compound (cisplatin and carboplatin) plus a second active cytotoxic agent, potentially in combination with bevacizumab, are preferred as the initial treatment for younger patients with advanced NSCLC and a good performance status (PS). For patients who are not candidates for a platinum containing regimen because of potential toxicity, regimens that may offer similar benefit based upon results of randomized trials are gemcitabine plus either docetaxel or paclitaxel or pemetrexed or vinorelbine and paclitaxel plus vinorelbine is described.

However, TPT-based combination regimens can also be used as the first-line therapy in the treatment of advanced NSCLC in patients who cannot tolerate platinum-based therapies because of the associated toxicity, multiple co-morbidities or poor functional status of the patient. TPT-based combination regimens have demonstrated promising antitumor activities with favorable toxicity profiles in such patients. Single-agent TPT may be particularly appropriate for patients in the second-line setting, in which palliation of symptoms is an important outcome of chemotherapy.

Several clinical trials have been conducted with TPT as a single agent in either IV or oral formulation for the treatment of NSCLC as a first or second-line treatment. These trials showed a response rate of 0-25% with a median survival time of 26 - 41 weeks [6-12]. TPT in combination with other chemotherapeutic agents like gemcitabine, vinorelbine and paclitaxel had superior antitumor activity compared with studies using single-agent TPT.

Here we reviewed all the clinical trials done with TPT to date in the treatment of NSCLC both as a single-agent and combination therapy.

## Phase I Clinical Trial of TPT as Single-Agent Therapy

Masuda et al [13] performed a phase I trial of TPT for the purpose of determining the maximum tolerated dose (MTD), pharmacokinetics and the dose limiting toxicity (DLT) of TPT when administered weekly to patients with advanced NSCLC. Twelve patients with stage IIIB or IV disease were treated with TPT by 30-min IV infusion on days 1, 8, and 15 every 4 weeks. The dose was escalated in 2 mg/m^2^ increments from the starting dose of 4 mg/m^2^ until the MTD was reached. After the MTD had been reached in previously treated patients, chemotherapy-naive patients were enrolled for treatment at that dose, and the dose was escalated to estimate the MTD in the treatment-naive group. This study showed that the recommended dose of TPT for phase II studies in previously untreated patients is 6 mg/m^2^ on days 1, 8, and 15, every 28 days, and 4 mg/m^2^ appears to be a suitable dose for use in previously treated patients with this schedule. This study also showed that weekly bolus infusion of TPT appeared to be more convenient for patients with mild toxicity profile compared to the FDA approved 5-day regimen.

## Phase I Clinical Trials of TPT as Combination Therapy

Raymond et al [14] in 1997 conducted a phase I study to determine the DLT of TPT in combination with cisplatin, to describe the principal toxicities, and to define the MTDs of the drugs in previously untreated patients with advanced NSCLC. The study was designed to evaluate escalated doses of TPT (starting at 0.75 mg/m^2^/day) as a 30-min infusion daily for five consecutive days with a fixed clinically relevant dose of 75 mg/m^2^ cisplatin given on day 1, every 3 weeks. This study showed that both severe neutropenia and thrombocytopenia precluded dose escalation of TPT and cisplatin administered on this schedule. In previously untreated patients, the first TPT/cisplatin dose level (0.75/75 mg/m^2^) was associated with intolerable myelosuppression, and, therefore, the dose levels evaluated in this study cannot be recommended for subsequent phase II investigations. The high toxicity of this schedule and the recent understanding of the pharmacokinetic interaction between those drugs may encourage the investigation of the alternate sequence of cisplatin after TPT in phase II studies.

Rinaldi et al [15] conducted a phase I-II trial of TPT and gemcitabine in patients with previously treated advanced NSCLC. It was done to determine the MTD, DLT, toxicity profile, and antitumor activity of TPT and gemcitabine combination therapy. This combination was relatively well tolerated and exhibited promising antitumor activity in patients with advanced, previously treated NSCLC.

Stupp et al [16] performed a phase I/II trial in patients with recurrent or metastatic NSCLC, where the patients were treated with IV TPT and IV vinorelbine every 21-day cycles, avoiding platinum. This study concluded that the combination regimen of TPT and vinorelbine is feasible for outpatient administration and is well tolerated with less toxicity than platinum-based regimens. Preliminary response data demonstrated good antitumor activity, suggesting that this regimen could make an excellent palliative treatment for advanced NSCLC.

Guarino et al [17] designed a phase I trial to determine the optimal dose of combination TPT, cisplatin, and gemcitabine in advanced NSCLC patients. This study showed that a 28-day cycle of TPT (1.75 mg/m^2^ on days 1, 8, and 15), cisplatin (20 mg/m^2^ on days 1, 8, and 15), and gemcitabine (1,000 mg/m^2^ on days 1 and 15) was a safe and well-tolerated outpatient treatment for advanced NSCLC.

Dabrow et al [18] performed a phase I/II trial of TPT combined with gemcitabine in patients with metastatic or unresectable NSCLC based on preclinical data showing *in vitro* synergy against an established lung adenocarcinoma cell line. The aim was to determine the MTD of TPT when the gemcitabine dose is held constant, as well as the DLT of this combination in NSCLC patients. The combination of gemcitabine and TPT seemed to be active against NSCLC with acceptable hematologic toxicity and minimal non-hematologic toxicity. The recommended dose for further study was 1,250 mg/m^2^ of gemcitabine (days 1, 8, and 15) and 2.0 mg/m^2^ of TPT (days 1, 8, and 15) administered every 28 days.

Beldner et al [19] conducted a phase I dose escalation study of vinorelbine and TPT combination therapy in patients with recurrent SCLC and NSCLC. The aim of this study was to evaluate the optimal dosage and the MTD of TPT and vinorelbine administered on an alternate dosing schedule. This study showed that vinorelbine and TPT administered on days 1 and 8 every 21 days was well tolerated without any DLT seen with previously investigated TPT schedules. This doublet provided a potentially active non-platinum containing doublet for the treatment of patients with advanced SCLC and NSCLC. Vinorelbine and TPT should therefore be investigated in subsequent phase II studies at a dose of 20 mg/m^2^ and 4 mg/m^2^, respectively.

In summary, phase I clinical trials done on TPT had shown good disease responses with minimal toxicity in combination with gemcitabine and vinorelbine at a dose range of 0.75 - 4 mg/m^2^/day given as infusion for 5 days every 3 weeks or as weekly bolus infusion for 3 weeks every 28 days [15, 16, 18, 19]. TPT in combination with cisplatin is associated with high toxicity [14]. TPT as a single agent had also shown good disease response with an MTD of 6 mg/m^2^ and 4 mg/m^2^ IV on days 1, 8, and 15, every 28 days in previously untreated patients and previously treated patients respectively [13]. The efficacy and safety of these doses are further studied in phase II clinical trials.

Table 1 displays all the above mentioned phase I clinical trials done on TPT in patients with advanced NSCLC as a combination therapy.

**Table 1:** Phase I Clinical Trials of Topotecan as Combination Therapy

Author and year	Phase	Patients	n	Drugs and dosage	Disease response	Median survival
Raymond et al, 1997 [14]	I	Untreated advanced NSCLC	14	TPT (0.75 mg/m^2^/day) as 30 min infusion daily for 5 days with cisplatin given at 75 mg/m^2^ on day 1 every 3 weeks	4 (30.7%) had PR	Tumor response lasted for 12 weeks
Rinaldi et al, 2001 [15]	I/II	Previously treated advanced NSCLC	19	TPT (0.75 mg/m^2^/day) as 30 min infusion daily for 5 days and gemcitabine 400 mg/m^2^ on days 1 and 5 only	3 (18%) had PR, 6 (32%) had SD	10 months
Stupp et al, 2001 [16]	I/II	Recurrent or metastatic NSCLC	29	IV TPT (0.5 - 1 mg/m^2^/day) for 5 days and IV vinorelbine (20 - 30 mg/m^2^/day) on days 1 and 5 only every 21 days	42% clinical response rate	13 months
Guarino et al, 2002 [17]	I	Untreated stage IIIb/IV NSCLC	30	IV TPT (1.75 mg/m^2^), cisplatin (20 mg/m^2^) on days 1, 8, and 15 and gemcitabine (1,000 mg/m^2^) on days 1 and 15 of a 28-day cycle	11 (38%) had PR	38 weeks. One-year survival rate was 33%.
Dabrow et al, 2003 [18]	I/II	Untreated stage IIIb/IV NSCLC	24	IV TPT (2 mg/m^2^) and gemcitabine (1,250 mg/m^2^) on days 1, 8, and 15 of a 28-day cycle	5 (21%) had PR	22 weeks
Beldner et al, 2007 [19]	I	Previously treated advanced NSCLC	18	IV vinorelbine (20 mg/m^2^) and TPT (2 - 4 mg/m^2^) on days 1 and 8 every 21 days	1 had PR, 4 (27%) had SD	10.5 months

PR: partial remission; SD: stable disease; n: number of patients.

## Phase II Clinical Trials of TPT as Single-Agent Therapy

Lynch et al [6] designed a phase II study to determine the clinical activity and toxicity spectrum of TPT in untreated patients with metastatic NSCLC. Toxicity included neutropenia and rash. They observed no objective clinical responses despite producing high-grade neutropenia. Phase II trials of TPT using different schedules or higher doses supported by growth factors may clarify the role of TPT in the treatment of NSCLC. However, the median survival time of 30 weeks was comparable with that obtained with combinations of agents in similar patient populations.

Perez-Soler et al [7] conducted a phase II study to assess the antitumor activity of TPT in patients with advanced NSCLC previously untreated with chemotherapy. Patients with stage IIIB or IV NSCLC with measurable disease in non-radiated fields were eligible. TPT at the dose and schedule mentioned in the above table had moderate antitumor activity in NSCLC; its activity was mostly limited to patients with squamous cell cancer. TPT was well tolerated, with myelosuppression of short duration being the most common and limiting toxicity.

Mainwaring et al [9] performed a phase II study to evaluate activity and toxicity of infusional TPT in patients with advanced NSCLC and advanced breast cancer who had not received previous chemotherapy for metastatic disease. The major toxicities were neutropenia and thrombocytopenia, with one episode of neutropenic sepsis. This study suggested that TPT delivered as a continuous IV infusion over 21 days as single-agent therapy does not appear to offer a clinical advantage over conventional 5-day schedules against advanced NSCLC and advanced breast cancer.

Kindler et al [10] conducted a phase II trial of TPT administered as a 21-day continuous IV infusion in previously untreated patients with stage IIIB and IV NSCLC. Although the major objective response rate was only 4%, patients treated with TPT given as a 21-day continuous IV infusion experienced a decrease in cancer-related symptoms and a 1-year survival of 39%.

Weitz et al [8] designed a randomized phase II trial of two different schedules of TPT in patients with advanced-stage NSCLC without prior cytotoxic chemotherapy. Patients were randomized to receive TPT at IV doses of 1.5 mg/m^2^/day over 30 min for 5 days every 3 weeks (arm A) or 1.3 mg/m^2^/day over 72 h every 4 weeks (arm B). The differences in time to progression and overall survival were not statistically significant. TPT has limited, single-agent activity in advanced NSCLC when given as 1.5 mg/m^2^/day over 30 min for 5 days every 3 weeks.

White et al [11] conducted a phase II trial to assess the activity of oral TPT in patients with advanced NSCLC previously untreated with chemotherapy. Oral TPT was administered at a dose of 2.3 mg/m^2^/day for 5 days every 21 days for up to six cycles unless disease progression or unacceptable toxicity occurred. Twelve patients (40%) experienced grade III/IV neutropenia. Five patients (16.6%) had grade III/IV anemia. There were two episodes of grade III/IV thrombocytopenia. Although oral TPT at the applied dose and schedule showed modest activity as a single agent, almost one-half of the patients had a stable disease, and median time to progression was 12.3 weeks. The overall median survival was a promising 39.9 weeks, and useful palliation of symptoms was seen.

Gonzalez et al [20] conducted a phase II study to determine the activity of TPT given at a dose of 1.25 mg/m^2^ IV for 5 days every 3 weeks in patients with advanced NSCLC pretreated with platinum and taxanes. This study showed that IV TPT at this dose and administration schedule displayed scant activity in terms of response rate in individuals with advanced NSCLC previously treated with platinum and taxanes.

In summary, phase II clinical trials done on TPT as single agent has not shown much efficacy except in the study done by Perez-Soler et al [7] which showed moderate antitumor activity at a dose of 1.5 mg/m^2^/day IV for 5 days every 21 days in previously untreated patients. Continuous IV infusion of TPT over 21 days did not appear to offer any clinical benefit as per the studies of Mainwaring et al and Kindler et al [9, 10]. Oral TPT at a dose of 2.3 mg/m^2^/day for 5 days every 21 days had also shown promising antitumor activity with palliation of symptoms in the study done by White et al [11]. The effectiveness of single-agent oral TPT has been further studied in a phase III trial.

### Adverse effects

The major adverse effects of TPT in phase I and II trials were hematologic characterized by anemia, leucopenia and thrombocytopenia in varying grades depending on the dose of TPT and the associated combination regimen. The non-hematologic adverse effects were characterized by lethargy, myalgia, rash, nausea, vomiting, constipation and catheter-associated infections.

Table 2 displays all the above mentioned phase II clinical trials done on TPT in patients with advanced NSCLC as a single-agent therapy.

**Table 2:** Phase II Clinical Trials of Topotecan as Single-Agent Therapy

Author and year	Phase	Patients	n	Dose of topotecan	Disease response	Median survival
Lynch et al, 1994 [6]	II	Untreated advanced NSCLC	20	2 mg/m^2^/day IV for 5 days every 21 days for two cycles	11 (55%) had SD, 9 (45%) had PRG.	7.6 months
Perez-Soler et al, 1996 [7]	II	Untreated advanced NSCLC	40	1.5 mg/m^2^/day for 5 days every 21 days	6 (15%) had PR, 10 had SD and 20 had PRG. 36% PR in patients with SCC.	38 weeks and 30% patients were alive at 1 year
Mainwaring et al, 1997 [9]	II	Untreated advanced NSCLC	12	0.6 mg/m^2^/day as continuous IV infusion for 21 days every 4 weeks	1 (8%) had PR.	Not reached
Kindler et al, 1998 [10]	II	Untreated advanced NSCLC	26	0.6 mg/m^2^/day as continuous IV infusion for 21 days every 4 weeks	1 (4%) had PR.	9 months and 1-year survival was 39%.
Weitz et al, 2000 [8]	II	Untreated advanced NSCLC	38	1.5 mg/m^2^/day over 30min for 5 days every 21 days	6 (16%) had PR.	257 days (37 weeks)
Weitz et al, 2000 [8]	II	Untreated advanced NSCLC	37	1.3 mg/m^2^/day as continuous infusion over 72 h every 21 days	3 (8%) had PR.	179 days (26 weeks)
White et al, 2000 [11]	II	Untreated advanced NSCLC	29	2.3 mg/m^2^/day orally for 5 days every 21 days for up to six cycles	13 (43.3%) had SD. No PR. 3 had radiological response.	39.9 weeks and 1-year survival of 33.3%
Gonzalez et al, 2011 [20]	II	Advanced NSCLC pre treated with platinum and taxanes	35	1.25 mg/m^2^/day IV daily for 5 days every 21 days for 73 cycles	1 (2.8%) had PR, 9 (25.7%) had SD, 23 (65.7%) had PRG.	70 days

SD: stable disease; PR: partial remission; PRG: progression of disease; SCC: squamous cell cancer, n: number of patients.

## Phase II Clinical Trials of TPT as Combination Therapy

Dowlati et al [21] designed a phase II trial of sequential topoisomerase I and II inhibition with TPT and etoposide in patients with advanced NSCLC. Hematologic toxicities included grade 4 neutropenia in 7% of patients. The authors noted that the lack of efficacy in their study may have been related to the short-lived (< 24 h) effect of topoisomerase II elevation after topoisomerase I inhibition; thus, it may be necessary to administer etoposide much earlier in a combined regimen with TPT.

Joppert et al [22] performed a phase II trial to determine the 1-year survival rate, efficacy and safety, produced by TPT and gemcitabine as first-line chemotherapy in advanced NSCLC. Grade 3 and 4 toxicities included neutropenia (53%), anemia (18%), thrombocytopenia (12%), asthenia (8%) and gastrointestinal disorders (8%); three patients (6%) experienced neutropenic fever. The combination of TPT/gemcitabine produced a 1-year survival similar to previous platinum-based regimens, when used as a first-line chemotherapy for advanced NSCLC. The toxicity profile was acceptable.

Lorusso et al [23] conducted a phase II trial where they used TPT plus ifosfamide in patients with advanced NSCLC. Anemia, neutropenia and thrombocytopenia were the major adverse effects. This study has shown antitumor activity with modest side effect profile and an overall disease control (PR + MR + SD). Nevertheless, the still low response rate and the shortness of median survival indicated the need for more effective second-line treatments in this disease.

Stathopoulos et al [24] conducted a phase II trial where they administered TPT and paclitaxel in patients with advanced NSCLC who were chemotherapy and radiotherapy naive. The major adverse effects were neutropenia and thrombocytopenia. This study showed that combination of TPT/paclitaxel in a weekly administration rendered a 40% response rate, with very low toxicity in stages IIIA, IIIB and IV NSCLC patients.

Jones et al [25] performed a randomized phase II trial to evaluate the efficacy of oral TPT compared with IV docetaxel in the second-line treatment of patients with NSCLC. Thirty-nine patients received 138 cycles of TPT. The overall response rate (ORR) was 8%, median time to progression (TTP) was 1.6 months, median survival was 8.4 months, and the 1- and 2-year survival rates were 36% and 13%, respectively, for patients receiving TPT. Oral TPT appeared to be active and well tolerated when administered as a fixed dose daily for 5 of every 7 days for 2 weeks every 21 days and might provide another treatment alternative for patients with advanced-stage NSCLC.

Powell et al [26] designed a phase II study to evaluate whether TPT in combination with bevacizumab improved progression-free survival (PFS) in patients with advanced, refractory NSCLC in a second-line setting. This study proved that bevacizumab in combination with TPT as a salvage therapy for metastatic NSCLC was well tolerated and worthy of further investigation.

In summary, phase II clinical trials done on TPT as combination therapy at the above mentioned doses had shown good antitumor activity in combination with gemcitabine, paclitaxel and bevacizumab with acceptable toxicity in previously untreated patients [22, 24, 26]. However, it has no benefit when combined with etoposide and ifosfamide [21, 23]. Oral TPT had also shown to have promising antitumor activity and well tolerated as a second-line treatment in a study done by Jones et al [25].

Table 3 displays all the above mentioned phase II clinical trials done on TPT in patients with advanced NSCLC as a combination therapy.

**Table 3:** Phase II Clinical Trials of Topotecan as Combination Therapy

Author and year	Phase	Patients	n	Drugs and dosage	Disease response	Median survival
Dowlati et al, 2001 [21]	II	Untreated advanced NSCLC	19	TPT at 0.85 mg/m^2^/day as a continuous 72-h infusion from days 1 to 3 and etoposide at 100 mg PO twice daily for 3 days on days 7 - 9. Total of 55 cycles.	1 PR and 2 SD	One-year survival rate was 33%.
Joppert et al, 2003 [22]	II	Untreated advanced NSCLC	51	TPT 1 mg/m^2^ on days 1 - 5, and gemcitabine 1 g/m^2^ on days 1 and 15 IV, every 28 days.	8 (17%) had PR, 11 (23%) had SD	7.6 months. One-year survival rate was 39%.
Lorusso et al, 2005 [23]	II	Previously treated advanced NSCLC	42	TPT (1.2 mg/m^2^) plus ifosfamide (1,200 mg/m^2^) IV for three consecutive days every 3 weeks. Total of three cycles.	6 (14.2%) had PR, 1 (2.4%) had MR, 14 (34%) had SD, 21 (51%) had PRG	26 weeks. One-year survival rate was 14%.
Stathopoulos et al, 2006 [24]	II	Untreated advanced NSCLC	45	TPT (1.75 mg/m^2^) infused over 30 min and paclitaxel (70 mg/m^2^) infused over 90 min, weekly for 3 weeks every 28 days up to three cycles.	2 (4.4%) had CR, 16 (35.6%) had PR, 21 (46.7%) had SD, 6 (13.3%) had PRG	9 months
Jones et al, 2008 [25]	II. RCT	Previously treated advanced NSCLC	80	39 received TPT (2 or 2.5 mg/day, for 5 of 7 days for 2 weeks) orally and 41 received docetaxel (75 mg/m^2^) every 21 days.	8% ORR with TPT and docetaxel	8.4 months with TPT and 7.6 months with docetaxel
Powell et al, 2013 [26]	II	Previously treated advanced NSCLC	42	TPT (4 mg/m^2^) on days 1, 8, and 15 and bevacizumab (10 mg/kg) on days 1 and 15 as IV infusion every 28 days.	14.3% had PR, 54.8% had SD, 28.6% had PRG	PFS was 5.1 months and overall survival was 11.5 months.

RCT: randomized controlled trial; ORR: overall response rate; MR: minimal remission; PFS: progression-free survival.

## TPT in Combination With Radiotherapy

Graham et al [27] designed a dose escalation clinical study with TPT and concurrent standard dose thoracic irradiation to assess its feasibility and toxicity in the treatment of patients with locally advanced, inoperable NSCLC. Twelve patients were entered in a prospective dose escalation trial and assigned to receive concurrent thoracic radiotherapy and TPT. The thoracic irradiation total tumor dose was 60 Gy in 30 fractions. Initial fields were to encompass the gross disease plus the mediastinum. TPT was delivered by bolus injection days 1 through 5, and days 22 through 26, beginning on the same day as the radiation therapy. The initial dose level was 0.5 mg/m^2^. Two additional dose levels of 0.75 mg/m^2^ and 1.0 mg/m^2^ were tested. At a follow-up of 12 - 24 months, two patients are alive and free of disease, three patients are alive with disease and the remaining seven patients died from the disease. This study showed that combination of TPT and thoracic radiotherapy for NSCLC, in the manner given by this protocol, could be safely given at a dose level of only 0.5 mg/m^2^ days 1 to 5 and 22 to 26 with 60 Gy of external beam radiotherapy. Higher doses of TPT were associated with high hematologic and gastrointestinal toxicity.

Patel et al [28] conducted a prospective phase II study of induction carboplatin and vinorelbine followed by concomitant TPT and accelerated radiotherapy (ART) in patients with locally advanced/unresectable NSCLC. Thirty-five patients received induction carboplatin on days 1 and 22, and vinorelbine (25 mg/m^2^) on days 1, 8, 22, and 29. During the concurrent chemoradiation, patients received IV TPT (0.5 mg/m^2^) on days 43 to 47, days 57 to 61, and days 71 to 75 before the morning radiotherapy (RT) fraction. RT was administered in an accelerated fashion at 2 Gy per fraction, twice daily for five consecutive days, every other week, to a cumulative dose of 60 Gy during a 5-week period. ORR was 71% (14% complete response, 57% partial response). Six of 35 (17%) patients had stable disease. Four (11%) patients progressed during treatment. The median survival was 17.9 months. This combined-modality regimen yielded encouraging overall survival rates, with no severe esophagitis. Using four-dimensional RT treatment planning, they planned to further evaluate altered fractionation RT and chemotherapy for this group of patients.

Seung et al [29] performed a phase II trial of combined modality therapy with concurrent TPT plus RT followed by consolidation chemotherapy for unresectable stage III and selected stage IV NSCLC. Twenty patients were treated with infusion TPT 0.4 mg/m^2^/day with three-dimensional conformal RT to 63 Gy both delivered Monday through Friday for 7 weeks. Patients without progression underwent consolidation chemotherapy with etoposide and a platinum agent for one cycle followed by two cycles of docetaxel. Eighteen patients had a partial response and one stable disease after induction chemoradiotherapy. The 3-year overall survival rate was 32% (median, 18 months). The local and distant PFS rates were 30% (median, 21 months) and 58% (median, not reached), respectively. Grade 3 hematologic toxicity was the major adverse effect. Continuous infusion TPT with RT was well tolerated and active in the treatment of poor-risk patients with unresectable stage III NSCLC.

In summary, studies done on TPT in combination with RT in patients with locally advanced stage III NSCLC shown to have mild improvement in survival rate with acceptable toxicity profile. However, further studies need to be done on this combination to prove the efficacy for its use in clinical practice.

## Phase III Clinical Trials of TPT

Ramlau et al [12] conducted a phase III study comparing oral TPT versus IV docetaxel in patients with previously treated NSCLC. Patients with stage III or IV NSCLC, PS ≤ 2, who had received only one prior chemotherapy regimen, were randomly assigned to treatment with oral TPT 2.3 mg/m^2^/day on days 1 to 5 or IV docetaxel 75 mg/m^2^ day 1 every 21 days. A total of 829 patients were randomly assigned. In intent-to-treat analysis, 1-year survival rates were 25.1% with TPT and 28.7% with docetaxel. Median survival was 27.9 weeks with TPT and 30.7 weeks with docetaxel. Although not statistically significant (log-rank P = 0.057), the higher survival rate with docetaxel was maintained across the entire treatment period. The median time to progression was 11.3 weeks with TPT versus 13.1 weeks with docetaxel. Grade 3 neutropenia occurred more frequently with docetaxel, grade 3 anemia and thrombocytopenia occurred more frequently with TPT. Oral TPT provided activity in the treatment of relapsed, locally advanced, unresectable NSCLC. Both regimens were well tolerated with differing safety profiles. TPT may provide an option for patients who desire an orally available treatment after relapse.

In summary, single-agent oral TPT at a dose of 2.3 mg/m^2^/day for 5 days every 21 days can be used as a second-line agent in patients with relapsed advanced NSCLC who desire oral therapy and where palliation of symptoms is desired. This is proven based on the trials done by Ramlau et al [12], Jones et al [25] and White et al [11].

## Conclusion and Approach to Therapy

Based on the clinical trials described above, TPT has demonstrated encouraging results in the treatment of NSCLC, a disease well known for its resistance to chemotherapy. Although ORRs with single-agent TPT have been relatively low in some studies, stable disease rates, when reported, were frequently high. As advanced NSCLC is a kind of disease where cure is not possible, delays in disease progression are desirable especially if toxicities are manageable. Therefore, single-agent TPT may be appropriate in the second-line setting.

TPT in combination with other antitumor agents has demonstrated superior antitumor activity compared with single agent. Some encouraging responses were obtained when IV TPT was combined with either gemcitabine or paclitaxel or bevacizumab or vinorelbine, with manageable toxicities at doses mentioned above in the clinical trials. In contrast, TPT in combination with etoposide or ifosfamide is associated with less antitumor activity and more adverse effects. The ideal regimen combining TPT and other agents has yet to be defined.

In conclusion, IV TPT in combination with other agents can be recommended as a first-line therapy for patients with advanced NSCLC who cannot tolerate standard platinum-based first-line treatments because of the associated toxicities, multiple co-morbid conditions or poor functional status. TPT has demonstrated response rates equivalent to those of standard therapy with manageable non-hematologic and hematologic toxicity when used as a combination therapy with other agents. TPT as a maintenance single agent in either oral or IV formulation can be used as a second-line therapy for patients who had progressed/relapsed or could not tolerate the standard therapies and where palliation of symptoms is desired.
